# Characterization of hexokinase gene family members in *Glycine max* and functional analysis of *GmHXK2* under salt stress

**DOI:** 10.3389/fgene.2023.1135290

**Published:** 2023-02-23

**Authors:** Shuai Chen, Zengyuan Tian, Yuqi Guo

**Affiliations:** ^1^ School of Life Sciences, Zhengzhou University, Zhengzhou, China; ^2^ School of Agricultural Sciences, Zhengzhou University, Zhengzhou, China

**Keywords:** Glycine max, hexokinase, gene family, VIGS-induced, salt tolerance

## Abstract

Hexokinase (HXK) is a bifunctional enzyme involved in carbohydrate metabolism and sugar signal sensing. HXK gene family has been extensively discussed in many species, while the detailed investigations of the family in *Glycine max* have yet to be reported. In this study, 17 *GmHXK* genes (*GmHXKs*) were identified in the *G. max* genome and the features of their encoded proteins, conserved domains, gene structures, and cis-acting elements were systematically characterized. The *GmHXK2* gene isolated from *G. max* was firstly constructed into plant expression vector pMDC83 and then transformed with *Agrobacterium tumefaciens* into *Arabidopsis thaliana*. The expression of integrated protein was analyzed by Western Blotting. Subcellular localization analysis showed that the *GmHXK2* was located on both vacuolar and cell membrane. Under salt stress, seedlings growth was significantly improved in *Arabidopsis* overexpressing *GmHXK2* gene. Furthermore, physiological indicators and expression of salt stress responsive genes involved in K^+^ and Na^+^ homeostasis were significantly lower in *GmHXK2*-silenced soybean seedlings obtained by virus-induced gene silencing (VIGS) technique under salt stress compared with the control plants. Our study showed that *GmHXK2* gene played an important role in resisting salt stress, which suggested potential value for the genetic improvement of abiotic resistant crops.

## 1 Introduction

Sugars play an important role during plant life, serving not only as the sources of energy and carbon structural components for growth and development but also as signaling molecules involved in many physiological processes such as germination (Kim et al., 2016), flowering (Zhang et al., 2020), senescence (Xiao et al., 2000), stomatal closure (Lugassi et al., 2015), and response to abiotic and biotic stresses (Sarowar et al., 2008; Granot et al., 2014; Lugassi et al., 2015). In higher plants, sucrose comprise the majority of carbohydrates produced by photosynthesis. Following synthesis, sucrose can then be directly stored and converted into hexose (glucose and fructose) by either invertase or sucrose synthase (Siemens et al., 2011; Granot et al., 2014). Afterwards, hexose is phosphorylated by hexokinase to produce hexose-6-P, which enters the glycolysis pathway and generates energy and intermediate metabolites involved in physiological activities of plants (David-Schwartz et al., 2013). HXK acts as a hexose sensor and signal in signaling networks to sense external nutrients, light, and hormones in addition to its role as a regulator of plant growth (Jang et al., 1997; Kim et al., 2013). Therefore, it has been recognized as a bifunctional enzyme and the key connecting element between sugar signaling and plant hormone signaling (Jang et al., 1997; Perata et al., 1997; Moore, 2004).

HXKs are encoded by a gene family in many plant species (Granot et al., 2014). *AtHXKs* genes were first isolated and identified from *A. thaliana* (Minet et al., 1992), revealing six members among which three proteins can phosphorylate hexoses, while the other three proteins lack catalytic activity (Karve et al., 2008). HXK gene family members have been systematically identified in various plant species. 10, 9, 4, 7, 11, 17 HXK genes members were identified respectively in *Oryza sativa* (Cho et al., 2006), *Zea mays* (Zhang et al., 2014), *Lycopersicon esculentum* (Kandel-Kfir et al., 2006), *Manihot esculenta* (Geng et al., 2017), *Physcomitrella patens* (Olsson et al., 2003) and *Gossypium hirsutum L* (Dou et al., 2022) in their genome.

Different HXKs are located in the membranes of various subcellular organelles such as the mitochondrion, chloroplast, cytoplasm, nucleus, Golgi, and vacuole (da-Silva et al., 2001; Olsson et al., 2003; Kandel-Kfir et al., 2006; Wang et al., 2014). Based on N-terminal amino acid sequences, plant HXK can be classified as Type A, Type B, or Type C (Giese et al., 2005; Karve et al., 2010). Type A HXKs has a plastid signal peptide structure containing a chloroplast transit peptide of about 30 amino acids, and it is mainly located in chloroplasts (Kandel-Kfir et al., 2006). Type B HXKs, which is mainly located in plastid (Olsson et al., 2003), contain an N-terminal hydrophobic membrane anchor domain of about 24 amino acids. Type C HXKs do not have signal peptides or a membrane anchor; they are cytosolic and seem to be only present in monocotyledonous plants and the moss *P. patens* (Karve et al., 2010; Cheng et al., 2011; Nilsson et al., 2011). As such, the diversity of HXK structure and subcellular localization lead to significant differences in their functions (Matschinsky et al., 2006; Riera et al., 2008).

Under salt stress, signaling substances such as ROS, ABA, Ca^2+^ in plants activate transcription factors through signal transduction, such as MYB, NAC, ERF, bZIP, etc., (Li et al., 2015; Wang et al., 2016; Baillo et al., 2019). The interaction between transcription factors and cis-regulatory elements promotes the expression of salt stress-responsive genes, inhibits the excessive accumulation of Na^+^ in plants and maintains the homeostasis of osmotic pressure. These include salt overly sensitive 1 (SOS1), high-affinity K^+^ transporter (HKT) and glutamate receptor-like channels (GLRs) located on the plasma membrane, sodium-hydrogen exchanger (NHX), cation/H^+^ exchanger (CHX and SALT3) and cyclic nucleotide-gated channels (CNGCs) located on the tonoplast and endomembranes, etc., (Plett et al., 2010; Guan et al., 2014; Ma et al., 2014). These genes are involved in the process of salt stress and regulate cation concentration to protect plants from damage caused by ion toxicity.

Soybean (*Glycine. max* L.) is the third most valuable plant crop worldwide as an oilseed crop (Bilal et al., 2020). However, the growth, production and quality of soybean are threatened by environment stress. With the increase in problems of soil salinity, salt stress has become one of the major stresses affecting growth and development of soybean (Leisner et al., 2017; Bilal et al., 2018). To date, little is known about any *G. max* HXK genes and their function in growth and development under abiotic salt stresses. In this study, we identified HXK gene family members of *G. max* based on the genome database. Next, we investigated the homology and phylogenetic relationship, gene structure, conserved motifs and cis-elements. The promoter sequences of most *GmHXKs* included many cis-elements including ABRE, ERE and LTR. It was supposed that *GmHXKs* were related with ABA signal pathway. Then, we assayed the expression pattern of *GmHXKs* under salt and drought stress, and found expression of *GmHXK2* in roots was increased gradually after treatment with NaCl or drought stress for 72 h. *GmHXK2* silenced plants obtained by VIGS technique were severely damaged after treatment with NaCl. We measured physiological indicators and the expression of some genes such as *GmSOS1*, *GmSALT3*, *GmHKT1*, *GmbZIP44*,etc., which were reported had a role in salt tolerance, in *GmHXK2* soybean silenced plants under salt stress conditions. Moreover, the DNA sequence of *GmHXK2* was cloned and constructed into the plant expression vector pMDC83, and it then transformed with *Agrobacterium tumefaciens* to *A. thaliana*. We analyzed the seed germination, plant growth and salt tolerance of homozygous T3 transgenic plants WT35 S:*GmHXK2*. Our study provided essential information concerning the physiological functions of *GmHXK2* and an important theoretical basis for genetic engineering soybeans to better tolerate to salt stress.

## 2 Materials and methods

### 2.1 Identification of the HXK gene family members in *G. max*

HMMER 3.0 software was used to search the soybean genome database for genes containing the structural domain of hexokinase (PF03727 and PF00349) and 17 soybean hexokinase genes were retrieved finally. The HXK gene sequences of *G. max* was downloaded from Ensembl Plants (http://plants.ensembl.org/index.html). The physical and chemical parameters of the proteins, including molecular weight (MV) and theoretical isoelectric point (PI), were computed by ExPASy (http://web.expasy.org/protparam/). The presence of a chloroplast transit peptide (cTP) in the protein sequence was predicted using the ChloroP 1.1 Server (http://www.cbs.dtu.dk/services/ChloroP/). The existence of transmembrane helices (TMHs) in the protein was predicted using TMHMM Server v. 2.0 (http://www.cbs.dtu.dk/services/TMHMM-2.0/). Chromosomal localizations of *GmHXKs* were mapped in Map Gene2Chromosome v2 (http://mg2c.iask.in/mg2c_v2.0/).

### 2.2 Multiple alignment, phylogenetic and expression pattern analysis

The sequences of the *GmHXKs* proteins obtained from the *G. max* genome were aligned using DNAMAN 7.0 software to search for conserved domains by inspection using sites present in *AtHXK1* as a reference. To compare evolutionary relationships, the putative HXKs from *G. max*, *A. thaliana*, *Solanum lycopersicum*, *O. sativa* and *Nicotiana tabacum* were used to construct the phylogenetic tree using MEGA-X with the neighbor-joining (NJ) method and 1,000 bootstrap replicates (Kumar et al., 2018). Expression data on *GmHXK* gene family members at different developmental stages and in different tissues under normal conditions were downloaded from the Soybase (https://www.soybase.org/). Data on differential expression for only 14 members were eventually obtained and used for subsequent analysis.

To analyze expression pattern of soybean seedlings under salt stress, soybean seedlings were grown in a growth chamber under greenhouse conditions of 28°C under a16-h light/8-h dark cycle. Three-week-old seedlings were treated with 0.5% NaCl (salt stress) or drought treatment (10%PEG 6000). The root samples of the seedlings were collected after treatment for 2-h, 8-h, 24-h, and 72-h. Then, different samples were frozen quickly in liquid nitrogen, and stored at −80°C for RNA extraction and analysis. Total RNA was isolated using the Plant RNA Kit (CWBIO, Beijing, China), and its concentration and purity were determined by Nanodrop2000 nucleic acid analyzer (Thermo, America). First-strand cDNA was synthesized from 0.5 µg of total RNA using the HiFi-MMLV cDNA Kit (CWBIO, Beijing, China), and then used as a template for qRT-PCR analysis using gene-specific primers (Supplementary Table S1). Data analysis of RT-qPCR was performed using 2^−ΔΔCT^ method (Livak and Schmittgen, 2001).

### 2.3 Gene structure, conserved motifs and cis-elements analysis

Gene structures were analyzed using Gene Structure Display Serve 2.0 (https://gsds.cbi.pku.edu. cn/) to investigate the exon-intron organizations of *GmHXK* genes based on their information given in the Ensembl Plants. The novel motifs of *GmHXKs* were searched using MEME (http://meme-suite. org/tools/meme) (Bailey et al., 2009). The parameters were set as follows: the site distribution was set to any number of repetitions (anr), the number of motifs was set to 10, and all other optional parameters remained default (He et al., 2019). The combination of gene structures, motifs, and phylogenetic tree was then generated using TBtools. In addition, the cis-acting regulatory elements in the 2000-bp genomic sequence upstream of the coding *GmHXK* gene sequences were investigated using the online PlantCARE databases. (http://bioinformatics.psb.ugent.be/webtools/plantcare/html/).

### 2.4 Construction of plant expression vector and transformation of arabidopsis

*GmHXK2* was obtained by PCR using the soybean genome DNA as a template with the primes, which are specific for *GmHXK2* gene. The PCR was performed using standard conditions: initial denaturation at 94°C for 2 min followed by 35 cycles of 94°C for 30 s, 56°C for 30 s and 72°C for 1 min, followed by a final extension of 72°C for 5 min. The PCR products of *GmHXK2* were gel purified using gel purification kit (TaKaRal MiniBEST Agarose Gel DNA Extraction Kit Ver.4.0) and used to construct the entry vector (Invitrogen, United States of America) by TOPO cloning reaction according to the manufacturer’s instructions. The entry vector containing the gene of the correct orientation and sequence was used to construct the target vector pMDC83 *via* the LR Clonase II enzyme mediated gateway cloning reaction according to manufacturer’s protocol. The recombinant plasmid pMDC83 with the hygomycin phosphotransferase gene under the regulation of double CaMV 35S promoter was generated, with GFP fused at the C-terminus of *GmHXK2*. Then, sequencing was performed to confirm the insertion into the correct gene in pMDC83. Next, the integrated vector was introduced into *A. tumefaciens* strains GV3101 by the liquid nitrogen freeze-thaw method. The *A. tumefaciens* transformation was transformed into WT Arabidopsis using the floral dipping technique. Transformed Arabidopsis plants were grown in a greenhouse under a 16/8 h light/dark cycle at 24/22°C with 70% relative humidity. Seeds harvested from the transformed plants (T0) were grown on 1/2 MS medium containing 20 mg L-1 hygromycin under the same growth conditions. Homozygous T3 progeny WT35S::*GmHXK2* derived from T2 population were selected and confirmed by PCR for further salt tolerance analysis.

### 2.5 Molecular analysis of transgenic *arabidopsis* plant and analysis on subcellular localization of *GmHXK2*

Genomic DNA of from the putative T3 transgenic Arabidopsis was isolated and analyzed by PCR amplification using specific primers. The amplification fragments were monitored in transgenic Arabidopsis with 35S:*GmHXK2*-GFP in pMDC83. No transformed seedlings were used as control.

The roots from 4-week-old seedlings expressing GFP in the transgenic Arabidopsis transformed with 2 × 35S::*GmHXK2*-GFP in pMDC83 were monitored using confocal laser-scanning microscopes. Images were captured at an excitation of 480 nm and emission between 515 and 565 nm for GFP. The subcellular localization of *GmHXK2*-GFP fusion proteins was confirmed.

### 2.6 Western blot analysis of transgenic arabidopsis plants

WT and WT35S::*GmHXK2* transgenic Arabidopsis plants were grown on 1/2 MS medium containing 20 mg L-1 hygromycin. After 15 days of cultivation, total proteins of the seedlings were extracted from WT and transgenic Arabidopsis seedlings with a buffer consisting of 50 mM Tris/HCl (pH 8.0), 150 mM NaCl, 1 mM EDTA, and 0.2% (w/v) Triton X-100, 4% β-mercaptoethanol, 1 mM dithiothreitol (DTT), and 1% (v/v) protease inhibitor cocktail of which were then used for protein quantification using the BCA Protein Quantitative Kit (Boster). The protein samples (200 μg amounts) were electrophoresed in 8% SDS-PAGE and the gels were transferred to nitrocellulose membranes. The membranes were blocked with TBST buffer (10 mM Tris/HCl, pH 7.5, 150 mM NaCl, and 0.05% Tween-20) supplemented with 5% non-fat milk for 2 h and incubated with primary antibodies (Anti-GFP antibody, abcam, diluted at 1:1,000) in TBST buffer with 5% BSA overnight at 4°C. Afterwards, the membranes were washed three times (10 min each) with TBST buffer and incubated with the secondary antibodies (Goat Anti-Mouse IgG H&L (HRP), abcam, dilution at 1:1,000) for 2 h. After washing three times with TBST buffer, the membranes were incubated with a chromogenic agent using the Enhanced HRP-DAB Chromogenic Substrate Kit (Boster).

### 2.7 Salt tolerance analysis of transgenic arabidopsis plants at the germination and seedling stage

Seeds of WT and WT35S::*GmHXK2* transgenic Arabidopsis plants of homozygous T3 generation were sterilized using 75% ethanol and 5% sodium hypochlorite for 1 min and 10 min, respectively. The seeds were washed six to ten times in sterilized water and were sown on half-strength MS (1/2 MS) medium with 0 and 100 mM NaCl. They were then transferred to a culture room after 3 days of vernalization at 4°C. Finally, root lengths and fresh weight of the germinated seeds were measured after planting for 7 days to detect salt tolerance in transgenic Arabidopsis overexpressing *GmHXK2.*

To analyze salt tolerance of transgenic Arabidopsis plants at the seedlings stage, treatment of plant samples were as followed. After the seeds were sterilized, they were sown on MS medium after 3 days of vernalization at 4°C, then they were transferred to a culture room and grown for 9 days. Next, Arabidopsis seedlings were transplanted into 1/2 MS medium containing 0 mM, 100 mM and 150 mM NaCl with or without 100 mM glucose (Glc). Finally, fresh and dry weight, length of the roots, malondialdehyde, chlorophyll and proline contents of the seedlings were measured after 6 days.

### 2.8 Determination of chlorophyll content

Chlorophyll was extracted using ethanol as solvent (Matschinsky et al., 2006). 100 mg of leaves was pulverized with 2 mL 95% ethanol, and the sample supernatant was measured with a spectrophotometer UV-1800PC at 665 nm and 649 nm. The chlorophyll extraction solution was calculated using the following formula:

Ca=13.95A665−6.8A649

Cb=24.96A6665−7.32A649

CT=Ca+Cb

The chlorophyll content per unit fresh weight of leaf was calculated using the following formula: Chlorophyll content (mg g-1) = (CT×extraction solution volume×dilution ratio)/(fresh  weight  of  leaves).

### 2.9 Determination of malondialdehyde (MDA) content

Fresh seedlings (0.1 g) were immediately homogenized with liquid nitrogen and were then mixed with 5 mL of 10% trichloroacetic acid (TCA) and centrifuged at 4,000 rpm for 10 min. The supernatant (2 mL) and 2 mL 0.6% thiobarbituric acid (TBA) were pipetted into a new tube. The mixture was incubated in a water bath at 100°C for 15 min and then cooled on ice and centrifuged at 5,000 rpm for 10 min. The absorbance of the supernatant was measured at 532 and 450 nm by spectrophotometer (Zhang et al., 2008). The MDA content was calculated using the formula: MDA (nmol g-1) = (6.45× 10^−6^×A532–0.56×10^−6^×A450)×V/W. V = volume of supernatant(L), W = weight of seedlings (g).

### 2.10 Determination of proline content

The proline content of Arabidopsis seedling was determined according to the method described by Jaemsaeng et al. (Zhang et al., 2008). Seedlings (0.05 g) were homogenized in 5 mL of sulphosalicylic acid (3%) and centrifuged for 10 min at 12,000 rpm. 2 mL of glacial acetic acid and 2 mL of acid ninhydrin were added to the supernatant (2.0 mL). The mixture was boiled in a water bath at 100°C for 30 min. After cooling, extraction was done with 4 mL of toluene. The absorbance was measured at 520 nm using toluene as a blank. The proline content (mg*g^-1^) = (Y × 5/2)/W. Y = content of proline in 2 mL supernatant. W = Weight of seedlings (g).

### 2.11 Determination of superoxide dismutase (SOD)

Seedings (0.1 g) were homogenized with 1 mL phosphate buffer (100 mM, ph = 7.8) and centrifuged at 12000 rpm for 30 min at 4°C. Then 0.1 mL of supernatant and phosphate buffer were added to the reaction solution (50 mM, ph = 7.8 Na_3_PO_4_,130 mM MET, 750uM NBT, 100uM EDTA, 20uM riboflavin) respectively. The reaction solution in which phosphate buffer was added served as the light control. The reaction was placed under fluorescent light for 15 min and then terminated in the dark. The absorbance value was measured at 560 nm. The SOD was calculated using the formula: SOD(U)=(OD_L_-OD_S_)*V/(0.5*OD_L_*V_S_*T*M). OD_L_ = Absorbance value of light control. OD_S_ = Absorbance value of samples. V = Total volume of sample extracts (ml). V_S_ = Volume of sample extract for determination (ml). T = Light reaction time (min). M = Weight of seedings(g) (Donahue et al., 1997).

### 2.12 Determination of electrolyte leakage (EL)

The conductivity of the sample (0.2 g) immersed in 20 mL of deionized water for 2 h at room temperature was measured as C1. Then the sample was boiled in a water bath for 15 min and cooled to room temperature. The conductivity measured was C2. Conductivity of deionized water as a blank control (C0). The electrolyte leakage (%)=(C1-C0)/(C2-C0) (Jungklang et al., 2017).

### 2.13 Construction of VIGS vectors and sprout infiltration for silenced plants

The specific fragments from CDS of *GmHXK2* and *GmPDS* were amplified using primers. The PCR products and the virus vector pTRV2 were digested with XbaI and BamHI(TAKARA),respectively. The products were ligated to obtain pTRV2-*HXK2* and pTRV2-*PDS* vectors. For VIGS experiment, plasmids of pTRV1, pTRV2 and pTRV2 recombinant vectors (pTRV2-*PDS* and pTRV2-*HXK2*) were transformed into *A. tumefaciens* strain GV3101 cells by using the freeze-thaw method. The empty plasmid pTRV2 was used as control (TRV:00). The *Agrobacterium strains* were inoculated into 50 mL of LB medium as above on a shaker at 180 rpm at 28°C for 12–16 h to an OD600 of 1.2–1.5. The *Agrobacterium* cells were centrifuged at 4000 *g* for 10 min at room temperature and washed twice, resuspended with the infiltration solution (10 mM MgCl_2_, 10 mM MES, and 200 μM acetosyringone) to a final OD600 of 0.8–1.0 and placed at room temperature in darkness for 3 h. The infiltration solution of the *Agrobacterium* strain containing pTRV1 was mixed with the infiltration solution of pTRV2 or Agrobacterium carrying the constructs in a 1:1 ratio (v/v) and 20–40 washed soybean seeds were added respectively (Zhao et al., 2020b). Soybean seeds that had been soaked for 24 h in darkness at room temperature were removed and planted in nutrient soil. The silencing efficiency of soybean seedlings was determined by qRT-PCR when the first true leaf was fully expanded.

### 2.14 Statistical analysis

The results of root length, fresh weights, and dry weights were from five independent experiments, and results of Chlorophyll, MDA, proline contents, EL and SOD were from three independent experiments. GraphPad Prism 5 was utilized for all statistical analysis. Data significance analysis was performed using Student’s t-test (Yang et al., 2022). Data are presented as mean ± SE. Single, double and three asterisks denote significant differences compared with the values of WT at *p* < 0.05, *p* < 0.01 and *p* < 0.001, respectively.

## 3 Results

### 3.1 Identification of *G. max* HXK gene family

In total, 17 HXK genes were identified in the *G. max* genome, and they were designated as *GmHXK1-17*. The name, Ensembl Plants accession number, mRNA and CDS length of nucleotide sequence as well as the length of amino acid sequence, MW, PI, chromosome location, cTP, and number of TMHs, are summarized in Table 1. As depicted in Table 1, the length of the CDS varied from 471 to 1,812 bp, encoding 156 to 504 amino acids and corresponding to molecular weights ranging from 17.42 to 55.40 kDa. The theoretical PIs of these proteins ranged from 5.18 to 8.76. There were three genes on chromosomes 1, 3 genes on chromosomes 17, and 2 genes on chromosomes 5, 7, 8, and 11. *GmHXK2*, *GmHXK10* and *GmHXK4* were distributed on chromosomes 9, 12 and 14, respectively. *GmHXK1*, *GmHXK2* and *GmHXK11* contained no cTP or TMHs. *GmHXK5*, *GmHXK6*, *GmHXK12–17* contained cTP and one TMH. *GmHXK3* and *GmHXK4* contained cTP but no TMHs.

**TABLE 1:** **Physicochemical property of HXK in *G. max*. CDS: coding sequences; MW (kDa): molecular weight (kilodaltons); PI: theoretical isoelectric point; cTP: chloroplast transit peptide; TMHs: transmembrane helices**.

Gene name	Gene ID	CDS Length (bp)	Amino acid sequence Length (aa)	MW(kDa)	PI	Chromosome location	cTP	Number of TMHs
GmHXK1	GLYMA_17G182400	471	156	17.42	5.18	17	none	0
GmHXK2	GLYMA_09G144600	891	296	32.92	6.22	9	none	0
GmHXK3	GLYMA_17G257800	1812	500	53.39	5.48	17	Yes	0
GmHXK4	GLYMA_14G218800	1,506	501	53.73	5.11	14	Yes	0
GmHXK5	GLYMA_05G110500	1,473	490	53.66	6.26	5	none	1
GmHXK6	GLYMA_17G156200	1,473	490	53.85	6.3	17	none	1
GmHXK7	GLYMA_01G226900	1,497	498	54.39	8.76	1	Yes	1
GmHXK8	GLYMA_11G015800	1,497	498	54.59	8.6	11	Yes	1
GmHXK9	GLYMA_11G095600	1,515	504	55.40	6.66	11	Yes	1
GmHXK10	GLYMA_12G021700	1,515	504	54.80	6.34	12	Yes	1
GmHXK11	GLYMA_07G015100	1,218	405	44.26	5.66	7	none	0
GmHXK12	GLYMA_05G226600	1,497	498	53.70	5.58	5	none	1
GmHXK13	GLYMA_01G007300	1,491	496	53.85	6.56	1	none	1
GmHXK14	GLYMA_08G200600	1,479	492	53.66	5.54	8	none	1
GmHXK15	GLYMA_08G033300	1,497	498	53.61	5.65	8	none	1
GmHXK16	GLYMA_01G007200	1,491	496	53.65	5.96	1	none	1
GmHXK17	GLYMA_07G124500	1,497	498	53.64	5.95	7	none	1

### 3.2 Multiple alignment and phylogenetic analysis of the *GmHXK* genes

**FIGURE 1:**
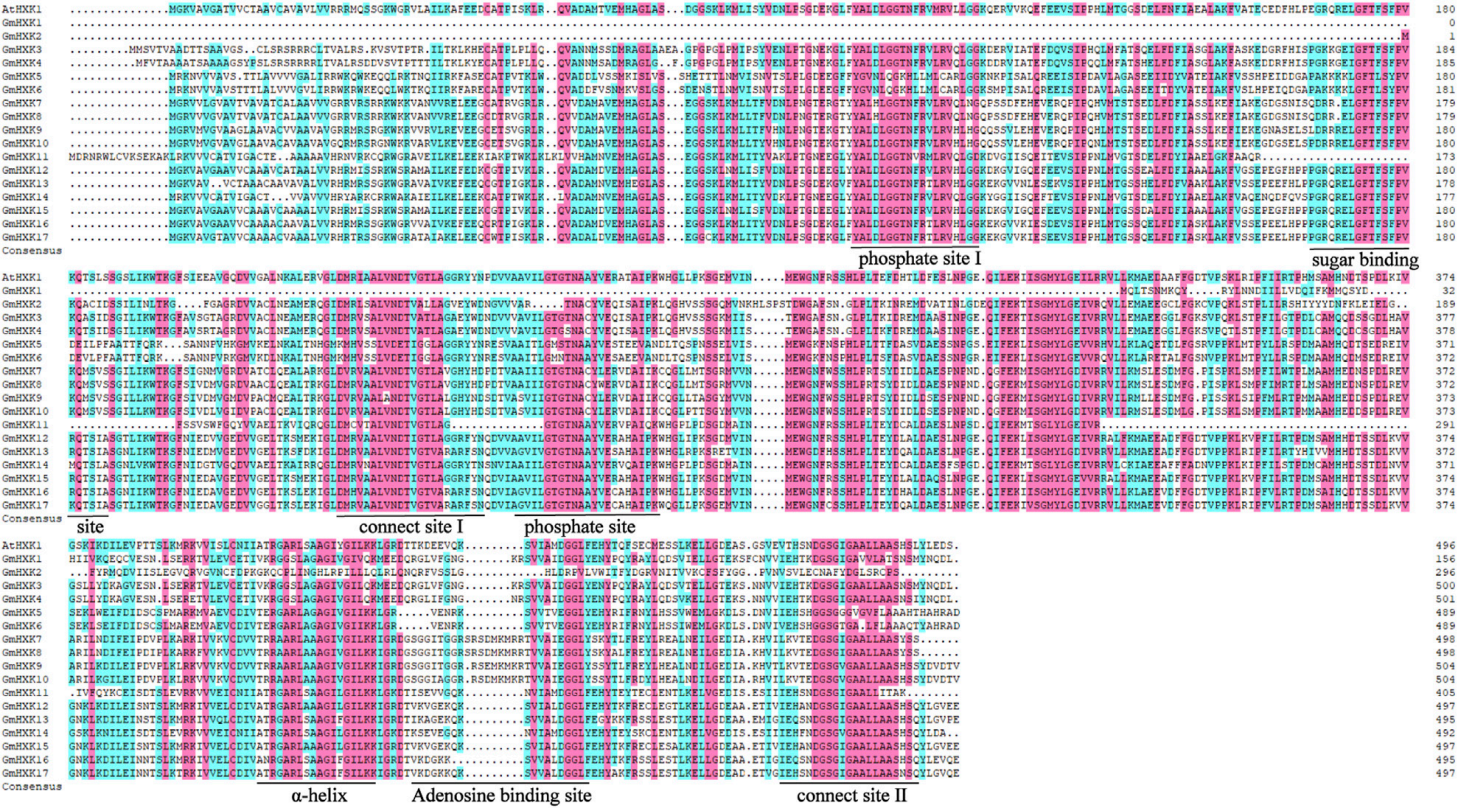
Multiple alignment of HXK amino acid sequences in *G. max*. Identical and similar amino acid residues are highlighted in black and pink shades, respectively. Core sugar binding site, phosphate sites (I and II), adenosine binding site, connection sites (I and II), and specific α-helix site are underlined.

To better study the structural difference between *GmHXKs* and their possible functional differences, the amino acid sequences of *G. max* were aligned and analyzed (Figure 1). Conserved domain analysis showed that most *GmHXKs* were multidomain proteins, which contained two phosphate sites (I and II), two connect sites (I and II), one α-helix site, one adenosine binding site, and one sugar binding site. However, *GmHXK*1 contained just three conserved domains, α-helix site, adenosine binding site, and sugar binding site. Additionally, *GmHXK2* contained phosphate site II, two connect sites (I and II), one α-helix site, one adenosine binding site, and one sugar binding site.

**FIGURE 2:**
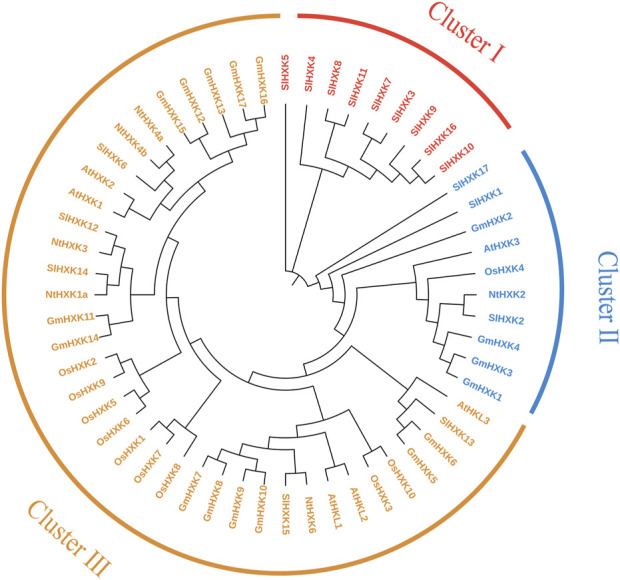
Phylogenetic analysis of the HXK gene family from *G. max* and other plant species. At, *A. thaliana*; Gm, *G. max*; Sl, *S. lycopersicum*; Os, *O. sativa*; Nt, *N. tobacum.* The amino acid sequences of HXK were aligned by Clustal X, and the tree was generated using the neighbor-joining method in MEGA-X with 1,000 bootstrap replicates.

To further reveal the evolutionary relationship of hexokinase family, the protein sequences of 56 hexokinases from five different plant species, including *G. max*, *A. thaliana*, *S. lycopersicum*, *O. sativa* and *N. tobacum* were used to construct phylogenetic tree using MEGA-X. As shown in Figure 2, all hexokinase genes were divided into three subfamilies (I–III). Most of the *HXK* genes of five different plant species were sorted into Cluster III. Cluster II contains fewer genes. It indicated the close relationship between the five plant species aforementioned. There were none *GmHXKs* in Cluster I, which contains only some hexokinase genes in *S. lycopersicum*.

### 3.3 Conserved motifs and gene structure analysis of *GmHXKs*

**FIGURE 3:**
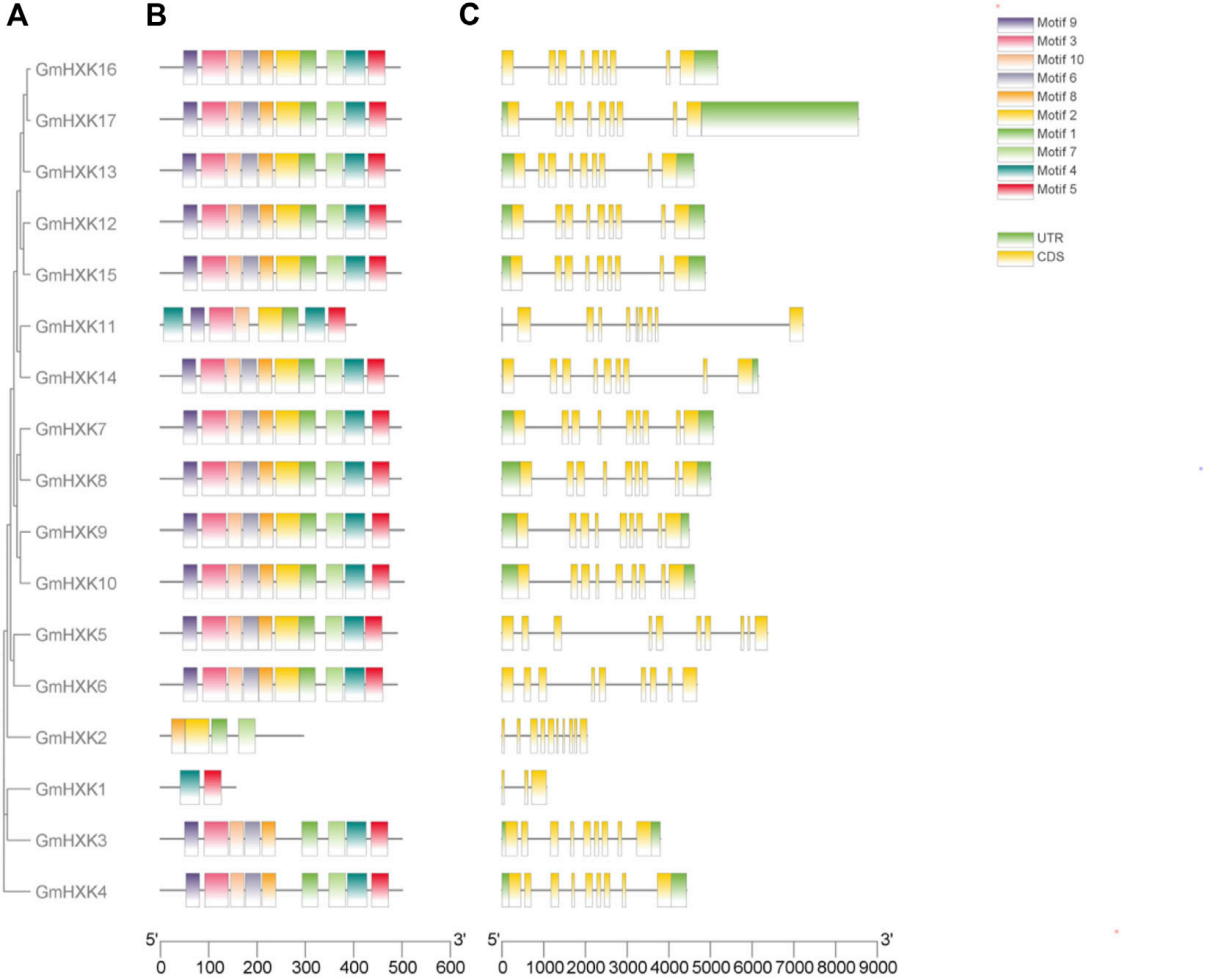
The conserved domain organization and exon-intron structure of *GmHXKs* genes. **(A)**. Phylogenetic relationships of *GmHXK* genes. The *GmHXK* amino acid sequences were aligned by Clustal X and the tree was generated using the Neighbor-Joining method in MEGA-X with 1,000 bootstrap replicates. **(B)**. The conserved domain analysis of *GmHXK* proteins using MEME. Different colors represent the different conserved motifs. **(C)**. Exon–intron structures of *GmHXK* genes. Exons and introns are shown as yellow boxes and black lines, respectively.

Generally speaking, the conserved regions of proteins frequently distinguish them from other proteins and determine the basis of their functions. To reveal the structural diversity and functional characteristics of the *GmHXK* family members, their conserved motifs were analyzed by MEME and mapped in the phylogenetic tree. In total, 10 conserved motifs were identified (Figure 3B). The detailed sequences and conserved motifs are shown in Supplementary Table S2. The identified motifs ranged from 29 to 50 amino acids in length. Among them, motifs 3, 6 and 8 were found to encode the hexokinase_1 domain, while motifs 1, 2, four and 5 encode the hexokinase_2 domain. Nevertheless, the functions of motif 7, 9 and 10 are unknown. *GmHXK*7-10, 12, 14–17 were found to have all 10 motifs, while other *GmHXK* family members had only portion motifs (Figure 3B). *GmHXK*3 and *GmHXK*4 contained all motifs except motif 2, and *GmHXK*5 and *GmHXK*6 contained all motifs except motif 9.

Different combinations of exons and introns can lead to diverse gene function. To explore the structure diversity of *GmHXK* genes, Gene Structure Display Server 2.0 was employed to analyze the distribution of exon-intron structure based on the corresponding genome and coding sequences (Figure 3C). The results showed that most *GmHXK* genes contained nine exons and eight introns. *GmHXK2* and *GmHXK*5 contained 10 exons and nine introns. *GmHXK*1, contained only three exons and two introns.

### 3.4 Cis-element analysis of the *GmHXK* genes

The identification of the cis-regulatory elements in the promoter part of the gene is important for functional and regulatory studies. To investigate the cis-regulatory elements of the 17 *GmHXK* genes, we analyzed the sequence 2,000 bp upstream of the start codon ATG. The cis-elements of the *GmHXK* genes were classified into three categories, involved in plant growth and development, stress responses and hormone-induced response (Supplementary Figure S1). The plant growth and development category contained seed-specific regulation (RY-element) and meristem expression (CAT-box) cis-elements. In the stress-responsive category, the elements included dehydration-responsive (DRE), anaerobic induction (ARE and GC-motif), low-temperature-responsive (LTR) and MYB-binding sites involved in drought inducibility (MBS), as well as defense and stress responsiveness (TCA). Seven types of phytohormone responsive cis-elements were detected, including auxin-responsive (AuxRR-core), abscisic acid-responsive (ABRE), methyl jasmonate-responsive (CGTCA-motif), ethylene-responsive (ERE), gibberellin responsive (GARE-motif), salicylic acid-responsive (TCA-element) and heat shock, osmotic stress, low pH, nutrient starvation-responsive (STRE). It is worth noting that the cis-elements MYB and MYC associated with phytohormone and abiotic stress were present in 17 *GmHXK* genes.

### 3.5 The expression pattern of *GmHXK* gene family

**FIGURE 4:**
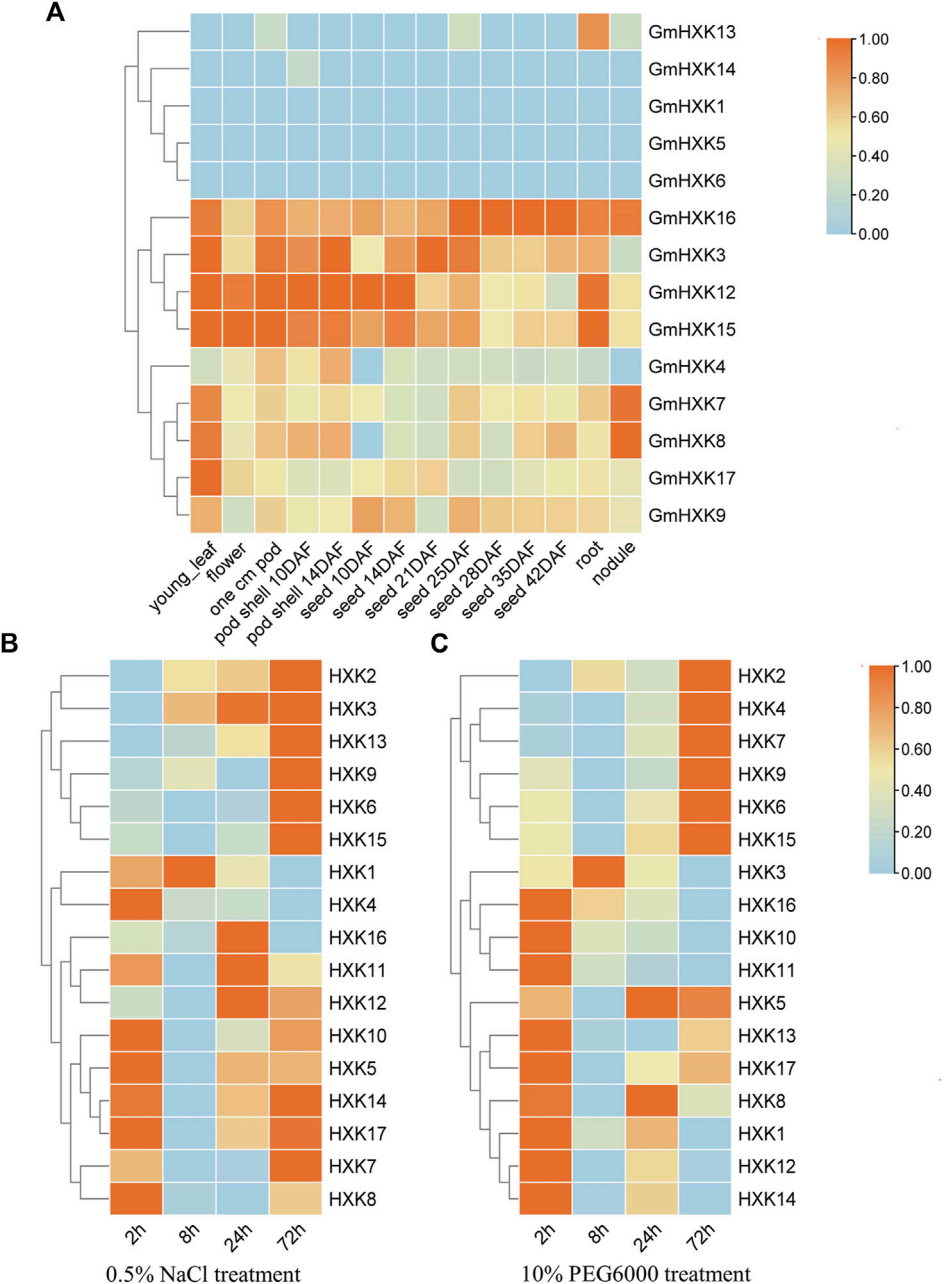
The expression pattern of *GmHXK* gene family. **(A)**. Tissue-specific expression profile of *GmHXK* under normal condition. Expression of *GmHXK* in roots under salt stress **(B)** and drought stress **(C)**. Data are presented as mean ± SE. Red indicates high expression levels blue indicates low expression levels.

We analyzed the expression patterns of 14 *GmHXK* family members in different tissues including young-leaf, flower, pod, pod shell, seed, root and nodule using data in soybase (Figure 4A). It showed that *GmHXK*1,5,6,13,14 was expressed at a very low level or not expressed in seven tissues above. The level of expression of *GmHXK*4,7,8,9,17 was not obvious in majority of the plant tissues except for that in a certain tissue. *GmHXK*3,12,15,16 were expressed obviously in most tissues.

And we analyzed also the expression of *GmHXKs* under salt and drought stress after treatments for different times by qRT-PCR. By analyzing expression data after treatment with NaCl or drought stress for 0, 2,8,24,72 h, we found that the expression of *GmHXK* 2,3,6,9,13,15 under treatment with NaCl (Figure 4B) and *GmHXK*2,4,6,7,9,15 under drought treatment with drought stress (Figure 4C) were increased after treatments within 72 h. The expression of other *GmHXKs* under salt or drought stress was obvious at the initial stage of treatment but decreased over time within 72 h. Owing to the increase in expression of *GmHXK*2,6,9,15 under both salt and drought stress, we selected *GmHXK*2 to study further its function under the salt stress.

### 3.6 Molecular characteristics of *GmHXK2*

**FIGURE 5:**
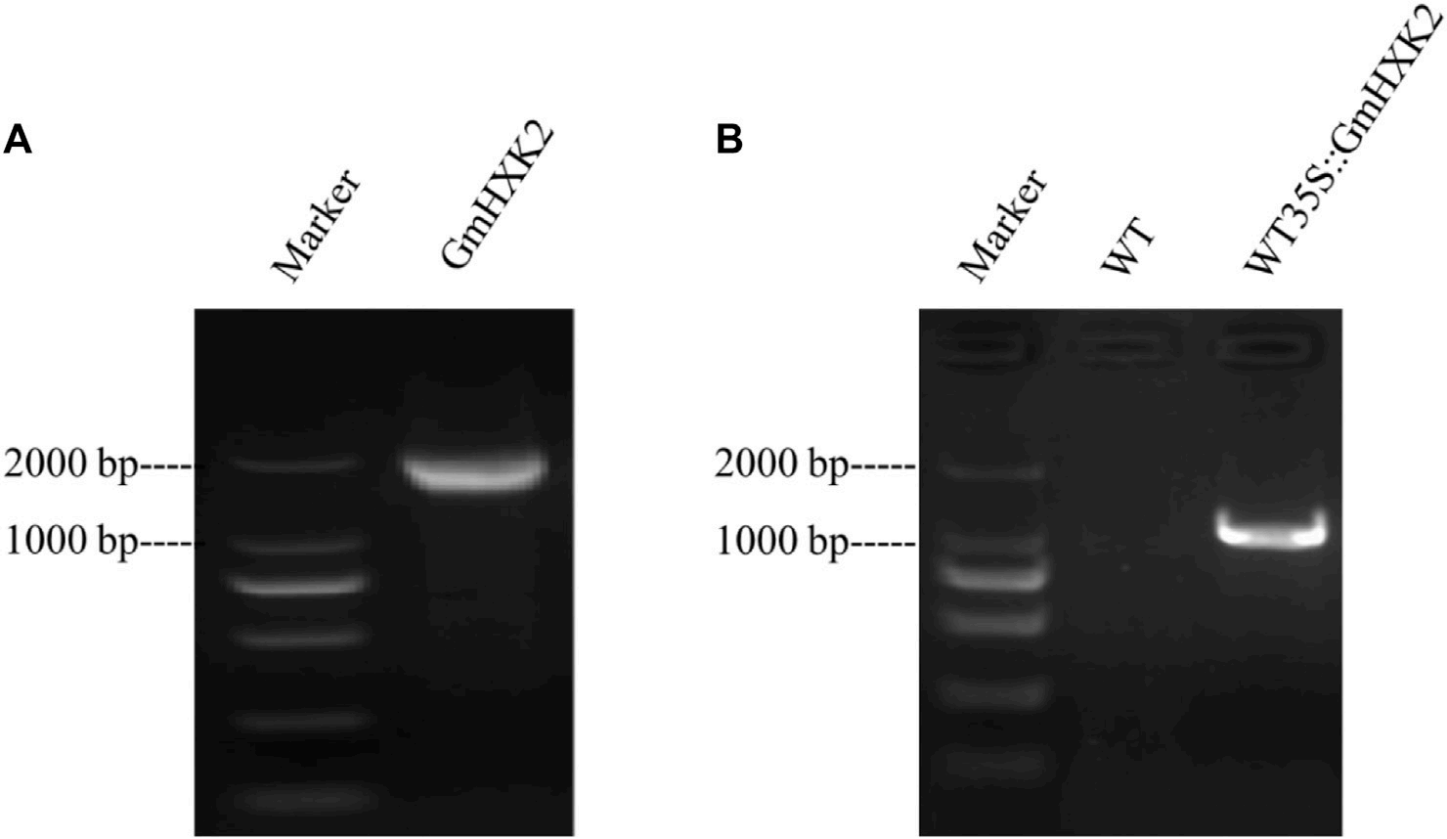
*GmHXK2* gene amplification and identification of transgenic Arabidopsis seedlings. **(A)**. PCR amplification of *GmHXK2* gene. Marker: DL 2000; **(B)**. PCR identification of transgenic Arabidopsis seedlings. Marker: DL 2000 (CWBIO, China).

*GmHXK2* gene (Gene ID: GLYMA_09G144600) fragment containing introns without stop codon, was isolated by PCR with primers using genome DNA as a template. Fragments of size 2,033 bp were detected (Figure 5A). Then, the PCR product was inserted into a pMD18-T vector and sequenced. Sequence analysis suggested that the fragment was identical to the known *GmHXK2* gene. The fragments were cloned into the entry vector TOPO by TOPO cloning reaction and subsequently into gateway destination vector pMDC83. Products amplified by PCR using genome DNA from T3 transgenic Arabidopsis WT35S::*GmHXK2* seedlings of expected size 1,357 bp were detected (Figure 5B). Our results show that the *GmHXK2* gene was integrated into the genome of WT.

### 3.7 Analysis on salt tolerance of *GmHXK2-*silenced plants

**FIGURE 6:**
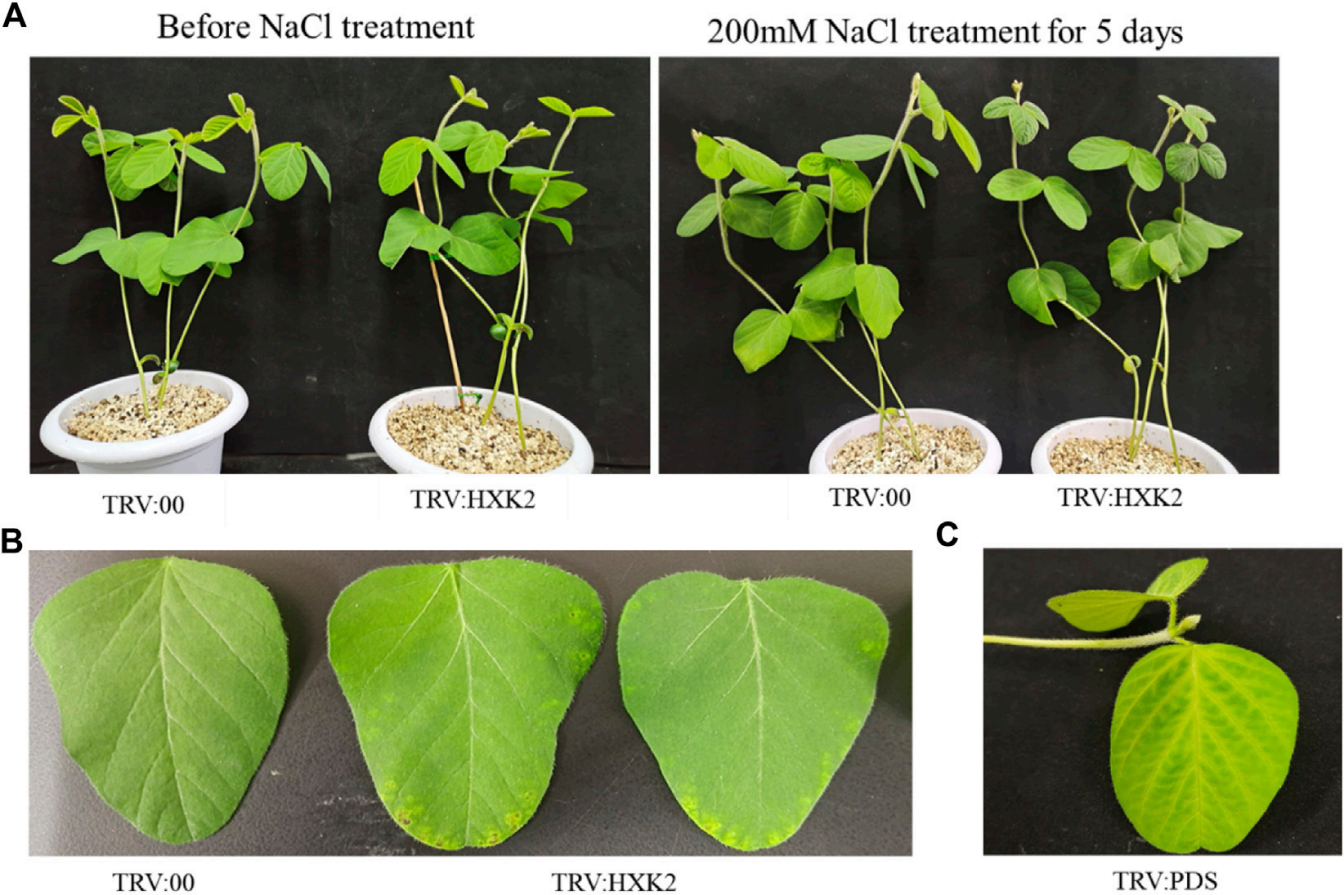
Phenotypic analysis of *GmHXK2*-silencing soybeans under salt stress. The seedings of TRV:00 and TRV:*HXK2* divided into two groups (control and salt treatment) were grown for 7 days on pot under normal condition, then the plants were treated with 0 or 200 mM NaCl for 5 days, respectively. Salt treatment group phenotype as shown in figure **(A–C)** Phenotypes of TRV:00, TRV:*HXK2* and TRV:*PDS*.

To further explore the role of *GmHXK2* in plant salt tolerance, we constructed a TRV-VIGS vector by selecting specific sequence in the CDS region of the *GmHXK2* gene and identified the efficiency in silencing of *GmHXK2-*silenced plants by qRT-PCR, which were 30%–50% of control plants (Supplementary Figure S2). The silenced plants showed different phenotype from the control. Margins of silenced plants in first true leaf were discolored and wilted (Figure 6B) compared with the control (TRV:00). The validity of silenced plants was further determined by the *PDS* phenotypes (Figure 6C). Finally, we selected the plants with the highest efficiency of silencing for the subsequent experiment on salt stress.

After treatment with salt stress, all leaves in soybean plants had been wilt, curled, and were yellow to some extent, and the leaves of *GmHXK2*-silenced plants were more severely damaged than control (Figure 6A).

**FIGURE 7:**
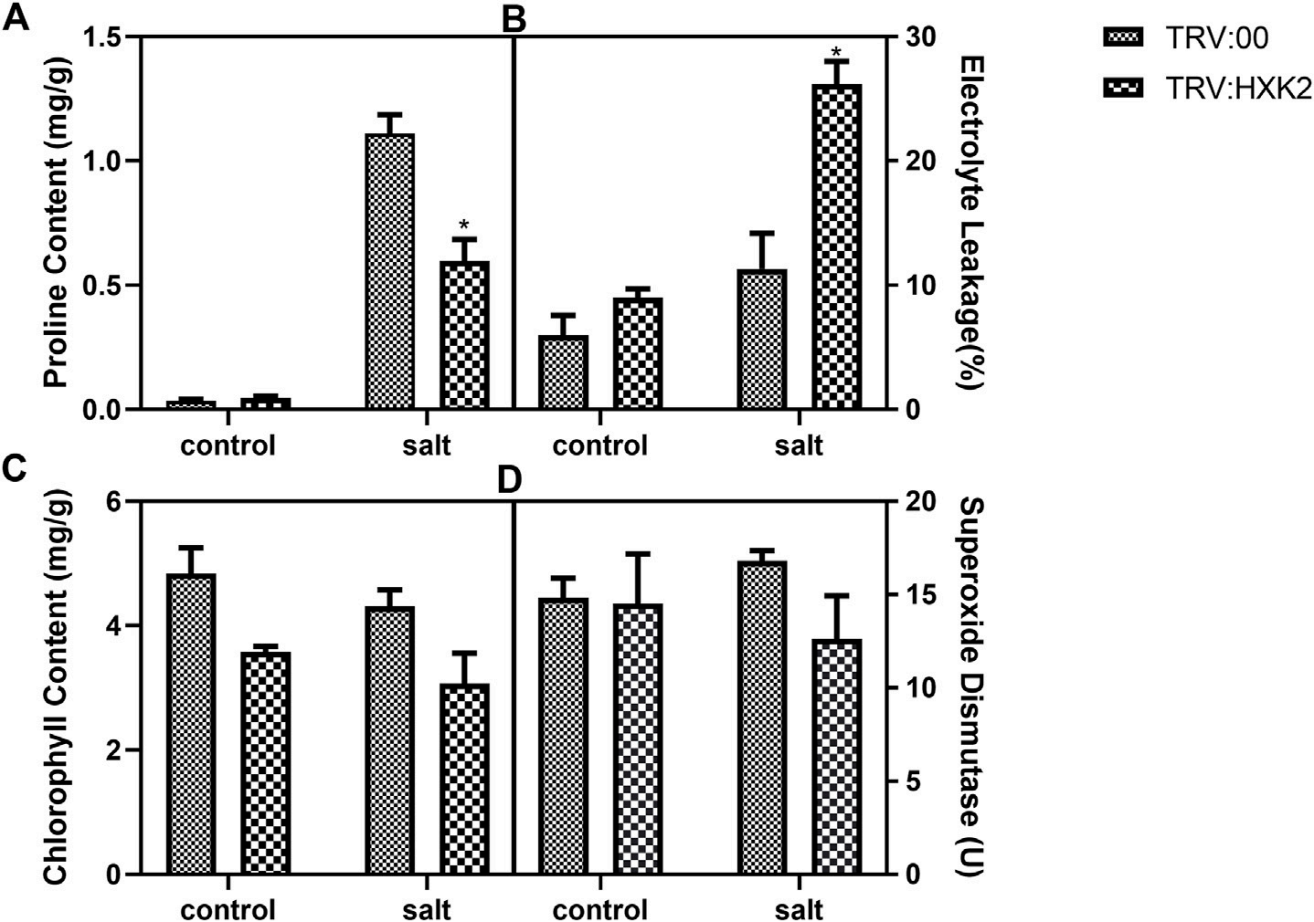
Physiological response of *GmHXK2* silent plant under salt stress. Content of proline **(A)**, EL **(B)**, chlorophyll **(C)** and SOD **(D)** were measured in TRV:00 and TRV:*HXK2* after treatment with 0 or 200 mM NaCl for 5-days. Data are presented as mean ± SE. Single asterisks denotes significant differences compared to the values of TRV:00 at *p* < 0.05; Student’s t-test.

To investigate the potential physiological mechanisms by which *GmHXK2* enhances plant tolerance, the proline, chlorophyll content, EL and SOD in TRV:00 and TRV:*HXK2* plants were measured under normal and salt stress conditions. Our results showed that the EL of TRV:*HXK2* (26.18%) was significantly higher than that of TRV:00 (11.33%) (Figure 7B) under salt conditions. This indicated that TRV:*HXK2* plants were damaged more seriously than the control plants. The content of proline, chlorophyll and SOD of TRV:*HXK2* (0.60 mg/g, 3.06 mg/g and 12.61 U, respectively) were decreased compared with that of TRV:00 plants (1.11 mg/g, 4.31 mg/g and 16.80 U, respectively) (Figures 7A, C, D). These results suggested that capability of TRV:*HXK2* plants in resisting salt stress and scavenging ROS generated intracellularly diminished compared with control plants.

### 3.8 Expression of Salt-Responsive genes in *GmHXK2-*silenced soybean plants

The plasma membrane Na^+^/H^+^ antiporters *GmSOS1* (salt overly sensitive) (Ma et al., 2020), the high-affinity potassium transporters (*GmHKT*s) (Sun et al., 2021), *GmbZIP44* (Zhao et al., 2020a), and *GmSALT3* as one of cation/H exchangers, which play a vital role in response to environmental salinity (Xu et al., 2022a), were known to be involved in the regulation of salt tolerance in soybean. So we investigated the expression of these Salt-Responsive genes to ascertain the relationship of *GmHXK2* and Na^+^ homeostasis.

**FIGURE 8:**
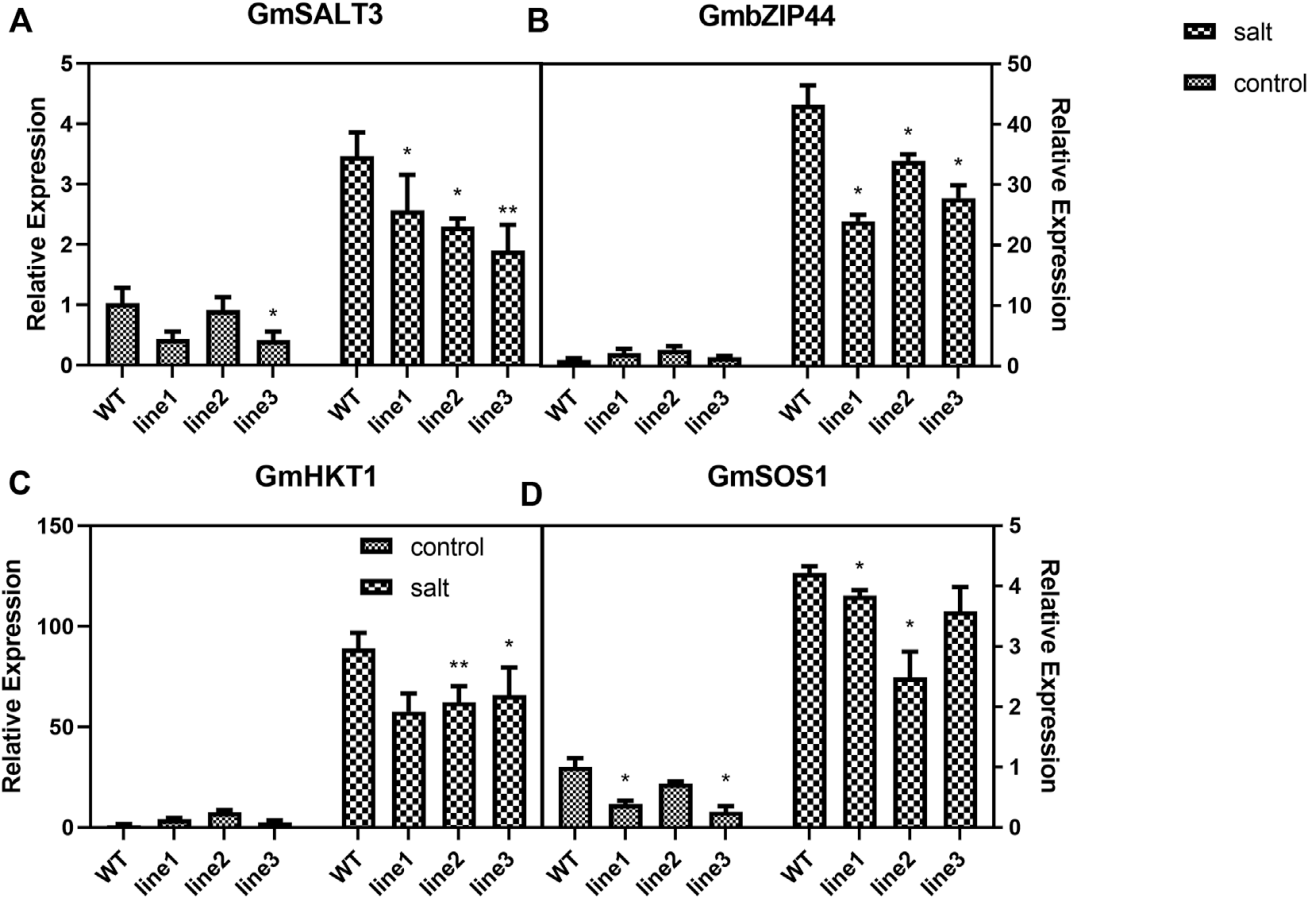
*GmHXK2* affect the expression of Salt-Responsive genes. **(A−D)**. Expression levels of salt stress-related genes. Data are presented as mean ± SE. Single and double asterisks denote significant differences compared to the values of WT at *p* < 0.05 and *p* < 0.01; Student’s t-test.

Under control conditions, expressions of both *GmSALT3* (Figure 8A) and *GmSOS1* (Figure 8D) were decreased in silenced plants compared with WT, while expression of *GmbZIP44* (Figure 8B) and *GmHKT1* (Figure 8C) were not significantly different. However, the expression of all four genes in *GmHXK2-*silenced plants under salt stress was decreased significantly than WT (Figure 8). These results suggested that silence of the *GmHXK2* gene downregulated the expression of salt stress-related genes involved in Na^+^ homeostasis, implying that *GmHXK2* had an important role in remaining homeostasis of Na^+^ and K^+^.

### 3.9 Subcellular localization and western blot analysis of transgenic arabidopsis plants

**FIGURE 9:**
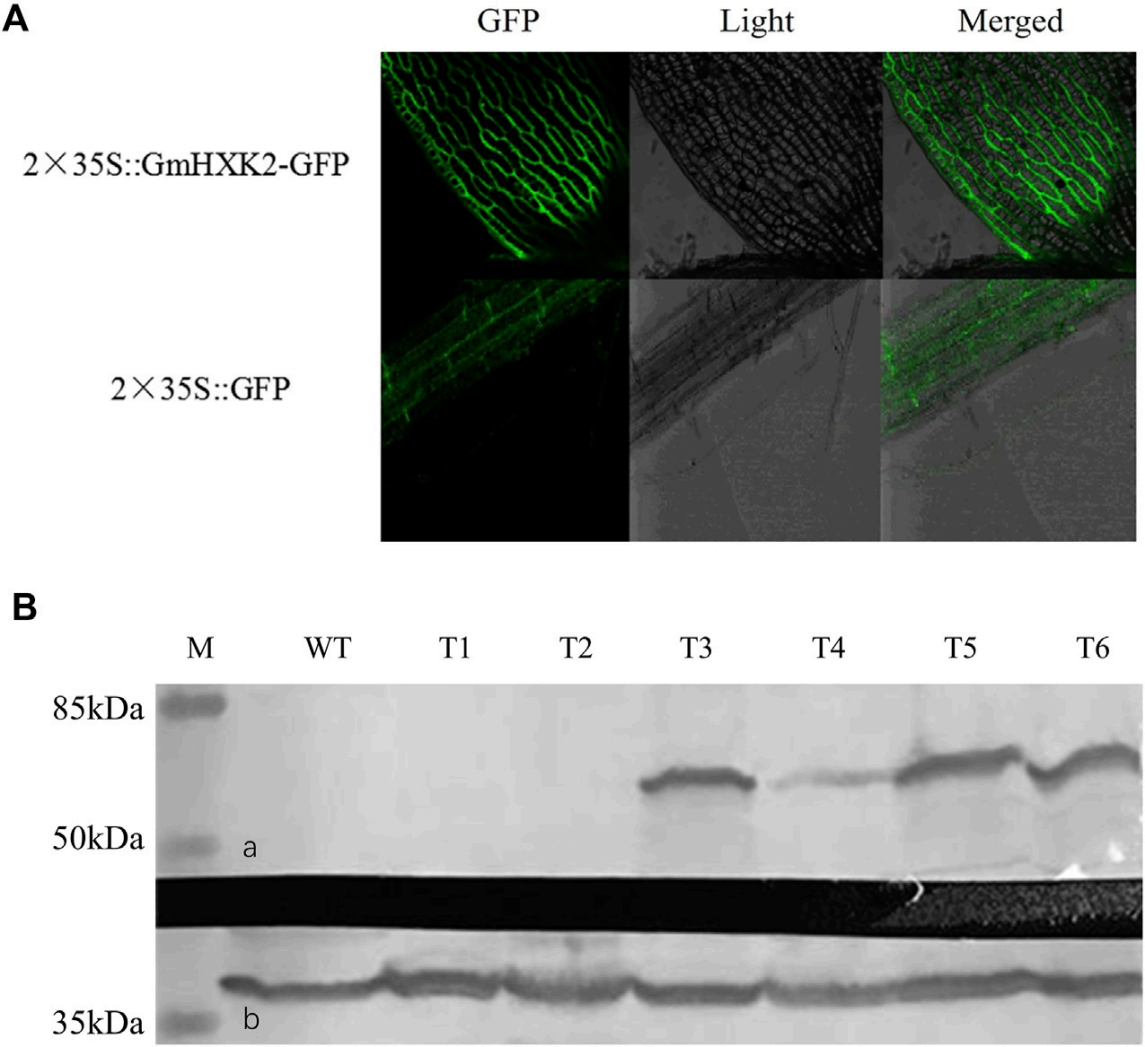
Subcellular localization and WB analysis of transgenic plants. **(A)**. Subcellular localization of *GmHXK2* in transgenic Arabidopsis root cells. **(B)**. Expression of *GmHXK2* in transgenic Arabidopsis detected by Western-blot analysis. **(A)**. *GmHXK2*-GFP fusion protein; **(B)**. GAPDH was used as internal control with molecular mass of 36 kDa (M: Marker; C: Wild type Arabidopsis; T1-T6: transgenic Arabidopsis plants.

As shown in Figure 9A, signals of *GmHXK2*-GFP were distributed on cell wall and cell membrane, which revealed that *GmHXK2* was predominantly localized on both vacuolar membrane and cell membrane. To examine *GmHXK2* protein expression, transgenic Arabidopsis seedlings WT35S::*GmHXK2* were further analyzed *via* immunoblotting with an antibody specific to GFP by Western blot. The results showed that T3-T6 transgenic lines expressed the integrated protein with an expected molecular mass of 59.92 kDa (Figure 9B), which corresponds to the fusion protein, while no proteins of this size were observed in that of WT seedlings.

### 3.10 Ectopic expression of *GmHXK2* promote the growth of *arabidopsis*

**FIGURE 10:**
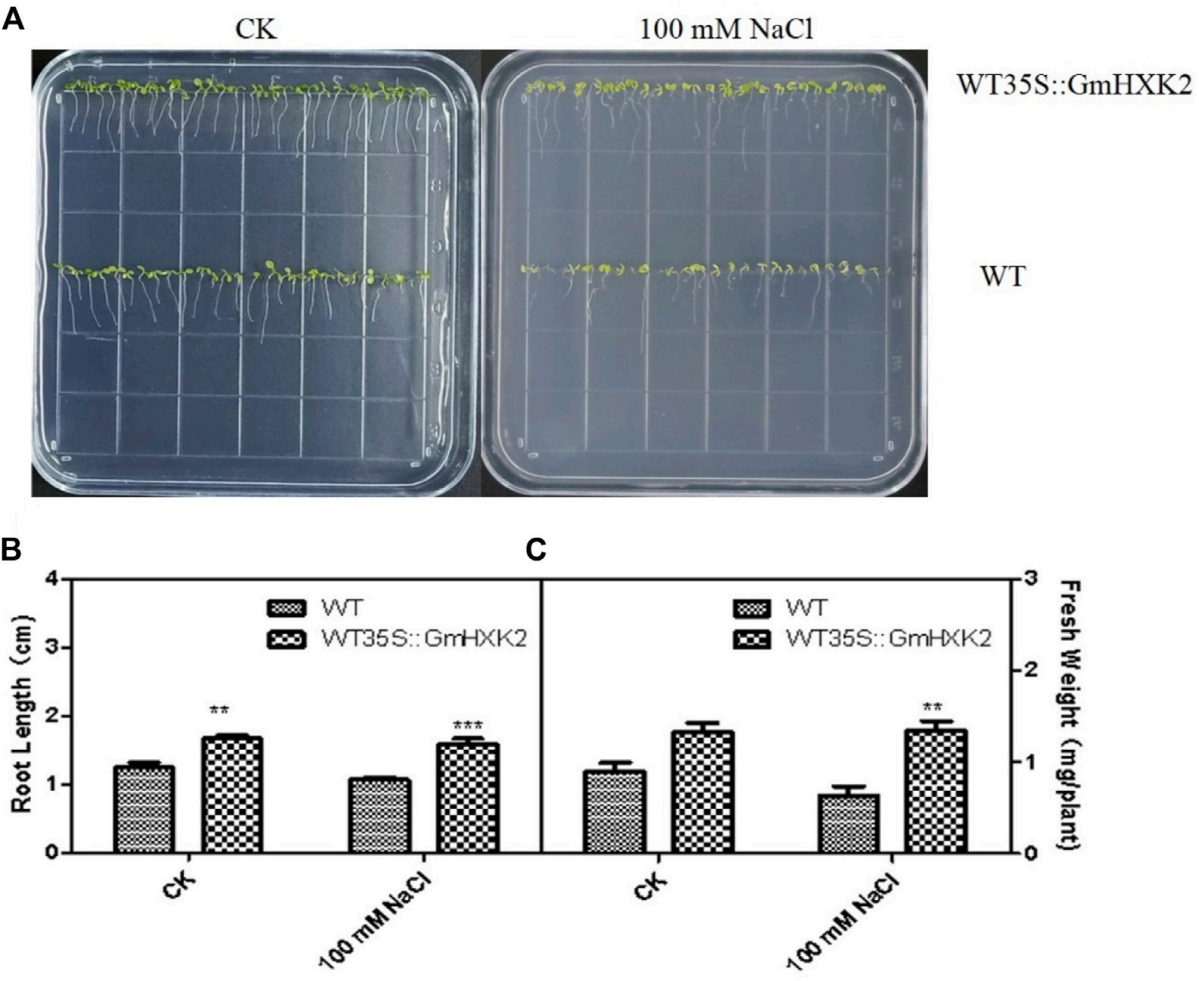
The phenotype **(A)**, root length **(B)** and fresh weight **(C)** of the WT and WT35S::*GmHXK2* transgenic Arabidopsis plants under salt stress at the germination stage. Seeds of WT and WT35S::*GmHXK2* were planted in 1/2 MS medium with or without100 mM NaCl for 7 days. Data are presented as mean ± SE (n = 5, biological replicates). Single, double and three asterisks denote significant differences at *p* < 0.05, *p* < 0.01 and *p* < 0.001, respectively; Student’s t-test.

As shown in Figure 10A, the WT and WT35S::*GmHXK2* transgenic Arabidopsis grew well in the control group. After 100 mM NaCl treatment, the growth of the Arabidopsis was inhibited, but the growth of WT was more suppressed than that of WT35S::*GmHXK2* transgenic Arabidopsis.

Under control condition, the root length of WT35S::*GmHXK2* was significantly higher than that of WT, and fresh weight of WT35S::*GmHXK2* were increased by 47% compared with WT. After NaCl treatment, the root length and fresh weight of Arabidopsis were decreased, and the root length and fresh weight of WT35S::*GmHXK2* were significantly higher than that of WT. (Figures 10B, C). Hence, the expression of *GmHXK2* promoted the growth of Arabidopsis.

### 3.11 Ectopic expression of *GmHXK2* enhance the salt resistance of *arabidopsis* at the seedling stage

**FIGURE 11:**
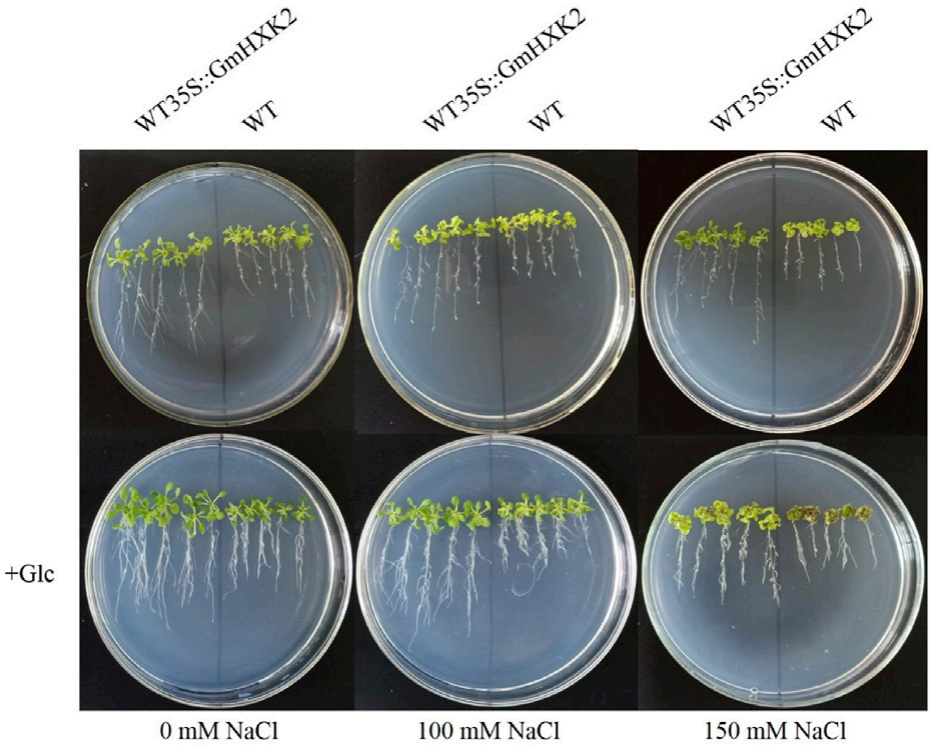
The phenotype of WT and WT35S::*GmHXK2* transgenic Arabidopsis plants under salt stress. Glc represents 100 mM glucose treatment.

To verify whether *GmHXK2*-expressing Arabidopsis contributes to salt tolerance, we observed the phenotype of transgenic Arabidopsis and WT after 6 days of NaCl and glucose treatments and then measured the physiology and biochemistry indexes. Under salt stress, the growth of Arabidopsis seedlings were obviously inhibited, the leaves of all plants gradually became yellow and shriveled in addition to roots becoming shorter. In addition, the growth of WT was more suppressed than that of WT35S::*GmHXK2*. After adding 100 mM glucose, the growth condition of seedlings was much better compared to those at 0, 100 and 150 mM NaCl treatments. (Figure 11).

**FIGURE 12:**
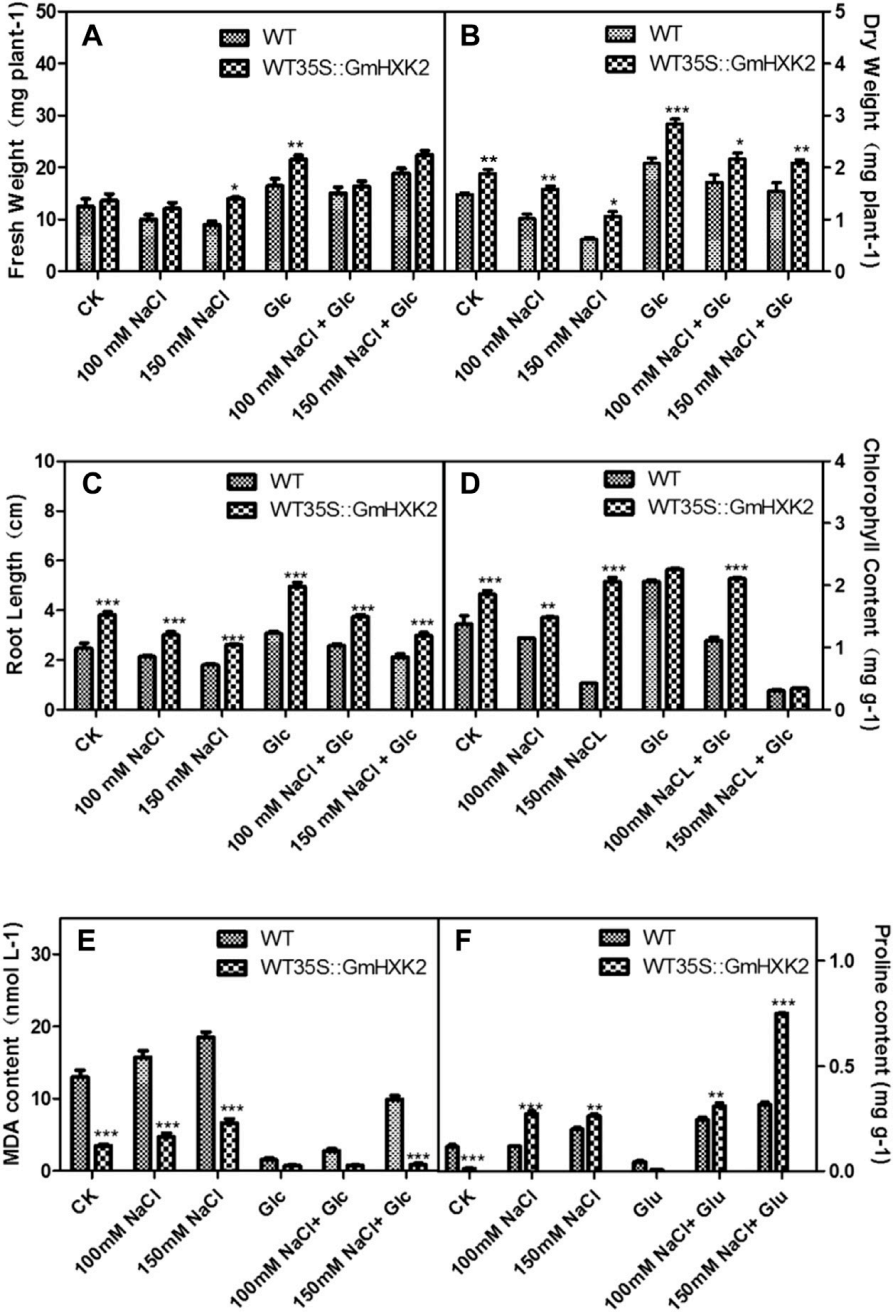
The fresh weight **(A)**, dry weight **(B)**, root length **(C)**, chlorophyll content **(D)**, MDA **(E)** and proline content **(F)** of the WT and WT35 S:*GmHXK2* transgenic Arabidopsis plants under salt stress. Data are presented as mean ± SE. Single, double and three asterisks denote significant differences compared to the values of WT at *p* < 0.05, *p* < 0.01 and *p* < 0.001, respectively; Student’s t-test.

After NaCl treatment, the fresh and dry weight, root length and chlorophyll content decreased, though the MDA and proline contents increased (Figure 12). As shown in Figures 12A, B, the fresh weight and dry weight of WT35S::*GmHXK2* transgenic Arabidopsis were higher than that of WT under 0, 100 and 150 mM NaCl treatment. After salt treatment, root length, chlorophyll and proline contents of WT35S::*GmHXK2* seedlings were significantly increased compared to WT, but the MDA content of WT35S::*GmHXK2* seedlings was much lower than that of WT (Figures 12C–F).

Exogenous glucose counteracted the effect on salt stress. The fresh weight and dry weight, root length, chlorophyll and proline contents of WT and WT35S::*GmHXK2* were increased compared to those at the 0, 100 and 150 mM NaCl stress. And the application of glucose under salt treatment inhibited the increase of MDA content under salt stress. (Figure 12).

These results revealed the expression of *GmHXK2* could enhanced the salt tolerance of plants during the seedling stage. In addition, 100 mM exogenous glucose alleviated the inhibition of salt stress on the growth of Arabidopsis plants.

## 4 Discussion

Hexokinases are very important enzymes in the growth and development of plants (Xiao et al., 2000). They not only play key roles in sugar signaling, but also involve in responding to abiotic stress in plants (Kim et al., 2013; Li et al., 2017). The *OsHXK10* gene in rice can regulate plant reproduction (Xu et al., 2008), the prunus HXK3 gene can promote tolerance to drought and salt stress (Perez-Diaz et al., 2021), expression of Arabidopsis *AtHXK1* in tobacco guard cells can attenuate transpiration in plants and improve salt and drought tolerance (Lugassi et al., 2019). In *Malus domestica* Borkh., *MdHXK1* interacts with the salt tolerance gene *MdNHX1* to improve the salt tolerance of plants (Sun et al., 2018). Though HXK genes have been widely studied in plants (Dai et al., 1999; Guglielminetti et al., 2000; Moore et al., 2003; Claeyssen et al., 2013; Li et al., 2017), HXK gene family members of *G. max* have not been characterized nor have their molecular properties under salt abiotic stresses been clarified until now.

Previous researches suggested that HXK proteins contain some conserved domains, phosphate 1 and 2, sugar binding site, and adenosine binding site, which are important to plant HXKs and essential for their enzymatic functions (Bork et al., 1993; Katz et al., 2000). In this study, a total of 17 HXK genes were identified in *G. max*. Except for *GmHXK*1, phosphate site, connect site, α-helix site, adenosine binding site, and sugar binding site, were well conserved in other *GmHXKs* (Figure 1). It suggested that they had the ability to phosphorylate hexoses. According to the characteristics of structure in Type A, B and C hexokinases and their distribution on subcellular organelle, we hypothesized that *GmHXK3*, *GmHXK4* and *GmHXK7-10* belong to Type A hexokinases and the other *GmHXKs* are Type B hexokinases using the data on the prediction of 17 hexokinase proteins and their subcellular localization in soybean.

ABA is a classical plant hormone whose level inside the plant is regulated by external environmental stress (Roychoudhury et al., 2013). Plants respond to environmental stress through interaction of transcription factors with a handful of cis-regulatory elements (Saidi and Hajibarat, 2019; Wang et al., 2021b). *GmHXK2* exhibited abscisic acid-responsive (ABRE), ethylene-responsive (ERE), and MYB elements, etc. ABRE is an eight bp cis-acting sequence present in the promoter region of most ABA-inducible or ABA-responsive genes. It has been shown that bZIP transcription factor genes bind to ABRE cis-acting elements to regulate stress-induced expression of related genes in many plants such as *A. thaliana* and soybean (Kang et al., 2002; Liao et al., 2008). MYB transcription factor family genes are widely distributed in plants, which act on MYB cis-elements that involve in many important physiological and biochemical processes, such as cell development, signal transduction, and stress response (Ambawat et al., 2013; Li et al., 2015). Ethylene response factor (ERF) plays an important role in plant stress tolerance by regulating gene expression through binding to the ERE element of stress response genes (Wang et al., 2016). Transgenic plants expressing the ERF gene were shown to have significantly increased tolerance to abiotic stresses such as drought and salt stress (Zhang et al., 2009; Rong et al., 2014). *GmHXKs* not only contains Cis-acting regulatory element involved in seed-specific regulation (RY-element), Cis-acting regulatory element essential for meristem expression (CAT-box) but also ABRE, ERE, MYB and MYC elements related with response to abiotic environmental stress, suggesting *GmHXKs* might be induced by different signal such as ABA and ethylene to take part in regulation of development and abiotic tolerance to environmental stress through being activated by MYB or MYC transcription factor.

VIGS is a very effective method for gene function analysis (Ramegowda et al., 2014). We obtained *GmHXK2*-silenced soybean plants by this method and investigated the growth and physiological parameters under salt stress. During the growth of plants, many physiological indicators are often used to verify the tolerance of plants (Du et al., 2018; Sun et al., 2019; Wang et al., 2019; Leng et al., 2021; Li et al., 2021). It has been demonstrated that the accumulation of proline under adversity conditions can help plants to resist abiotic stresses (Lehmann et al., 2010; Xu et al., 2022b). To a certain extent chlorophyll content can indicate the degree of plant tolerance to stress. The reactive oxygen species (ROS) are by-products produced by various metabolic pathways within plant cells. Plants produce a range of defensive substances to maintain the dynamic intracellular equilibrium and prevent from causing damage to the organism due to accumulation of ROS. SOD is an enzyme that scavenges ROS in plants, and its level reflects the plant’s tolerance to stress (Apel and Hirt, 2004; Mittler et al., 2004; Wang et al., 2021a). EL can indicate the degree of plant cell membrane damage, higher EL suggests a greater degree of cell damage (Jungklang et al., 2017). Based on the data on phenotype (Figure 5) and physiological indicators that we obtained (Figure 6), silence of *GmHXK2* decreased the salt tolerance of the plants, we deduced that the *GmHXK2* gene is contributed to salt tolerance in soybean plants under salt stress.

Under salt stress, excessive sodium (Na^+^) inhibits enzyme activity and disrupts potassium (K^+^) uptake, leading to decrease in ration of K^+^/Na^+^. Plants have evolved multiple physiological mechanisms to cope with external stresses (Zhao et al., 2020a). *GmHXKT1*, *GmSOS1*, and *GmSALT3* are genes that maintain ion homeostasis in cells in soybean plants. SOS1 is the key gene in the SOS pathway that regulates Na^+^ concentration and is believed to play an important role in osmoregulation (Shi et al., 2002; Olias et al., 2009; Ma et al., 2014). HKT is one of Na^+^/K^+^ transporters, which protects plants from Na^+^ accumulation in photosynthetic organs to improve the salt tolerance of plants by maintaining the Na^+^/K^+^ balance under salt stress (Berthomieu et al., 2003; Ren et al., 2005; Plett et al., 2010). *GmSALT3* encodes a protein of the cation/H^+^ exchanger family which has a role in sensing or coping with salinity (Guan et al., 2014). *GmbZIP44*, as stress-responsive protein in the ABA pathway, can binds to the ABRE cis-regulatory element of target genes to regulate downstream gene expression (Kang et al., 2002; Liao et al., 2008). In our findings, expression of *GmHXK2* gene were increased gradually in roots after treatment with NaCl for 2 h–72 h (Figures 4B, C). And the expression *GmHXKT1*, *GmSOS1*, and *GmSALT3* were downregulated in *GmHXK2*-silenced soybean plants after treatment with NaCl compared with WT (Figure 8). These results confirmed that the silence of *GmHXK2* in soybean plants was correlated significantly with decrease in expression of gene involving in uptake, transport and homeostasis of Na^+^, which reflected the important role of *GmHXK2* through regulating homeostasis between K^+^ and Na^+^ to improve salt tolerance of soybean plants.

Plant transcription factors can bind to specific cis-element through their conserved domain. For example, overexpression of *ThMYB8* decreased ROS levels and maintained K^+^/Na^+^ homeostasis (Liu et al., 2021). *TaPIMP1* can acts by bind to a few of MYB-binding sites to regulate of genes related with abiotic stress in response to ABA or salicylic acid signal (Zhang et al., 2012). According to our results, we supposed that in response to salt stress, MYB or MYC transcription factor might be induced by ABA or ethylene, afterwards they might bind to *GmHXK2* and triggered target genes downstream such as some genes related with homeostasis of Na^+^ to regulate the salt tolerance of soybean plants. The deduction on the molecular mechanisms of *GmHXK2* gene in salt tolerance need to be further confirmed by extensive experiments. Furthermore, *Arabidopsis* plants of ectopic expressing *GmHXK2* had the longer roots, lower levels of MDA contents, and higher levels of proline and chlorophyll content under salt stress, specially at conditions of exogenous application with glucose, indicating that *GmHXK2* might also sense and respond to glucose signal to participate in salt tolerance under salt stress.

## 5 Conclusion

In this work, we not only identified HXK gene family members but also obtained *GmHXK2* transgenic *Arabidopsis* lines and *GmHXK2-*silenced soybean plants. Subsequent analysis found that overexpressing *GmHXK2* in Arabidopsis plants improved their tolerance to salt stresses. Moreover, 100 mM of exogenous glucose can alleviate plant growth inhibition under salt stress. Conversely, *GmHXK2-*silenced soybean plants reduced the expression of salt tolerance genes, which lead to less tolerant to salt stress. Our results not only provided the theoretical foundation for further research on HXK gene family in *G. max*, but also they could provide important information for breeding stress-resistant *G. max* cultivars.

## Date availability statement

The original contributions presented in the study are included in the article/Supplementary Material, further inquiries can be directed to the corresponding author**.**
