# Supplementary material for: Characterization of hexokinase gene family members in Glycine max and functional analysis of GmHXK2 under salt stress
Source: Front Genet. 2023 Feb 23;14:1135290. doi: 10.3389/fgene.2023.1135290 (PMC9996050; doi:10.3389/fgene.2023.1135290)
Supplement: Supplementary file 1 [file DataSheet1.ZIP › Supplementary.docx]

Supplementary Material

# Supplementary Table

**Table S1.** List of primers used in the manuscript

**Table S2.** Optimal amino acid sequence and function of motif.

**Table S3.** The root length and fresh weight of the WT and WT35S::*GmHXK2* transgenic Arabidopsis plants under salt stress at the germination stage.

**Table S4.** The fresh weight, dry weight, root length, chlorophyll content, MDA and proline content of the WT and WT35S::*GmHXK2* transgenic Arabidopsis plants under salt stress.

**Table S5.** Physiological responses and silencing efficiency of *GmHXK2*-silenced plants under salt stress

**Table S6.** The expression pattern data of *GmHXK* gene family.

# Supplementary Figure

**Fig S1.** The cis-elements of HXK genes in *G. max*. The white-red scheme is labeled above the heat map; white and red represent 0 and higher number of cis-elements, respectively.

**Fig S2.** Identification of silencing efficiency of soybean silent plants (** p<0.01, *** p<0.001; Student's t‐test).
