# Supplementary figures and images for: Characterization of hexokinase gene family members in Glycine max and functional analysis of GmHXK2 under salt stress
Source: Front Genet. 2023 Feb 23;14:1135290. doi: 10.3389/fgene.2023.1135290 (PMC9996050; doi:10.3389/fgene.2023.1135290)

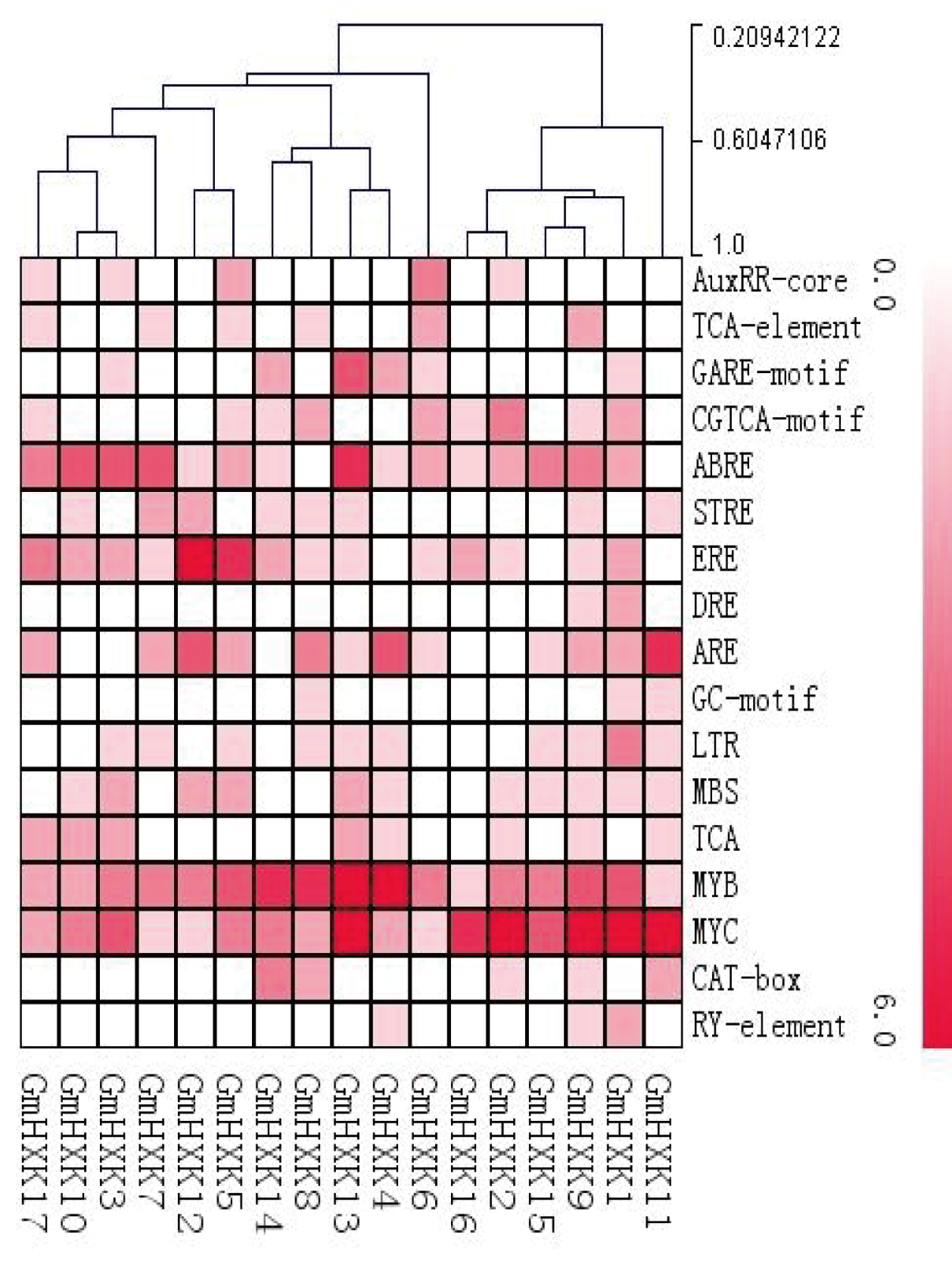

Supplement: Supplementary file 1 [file DataSheet1.ZIP › Fig S1.tif]

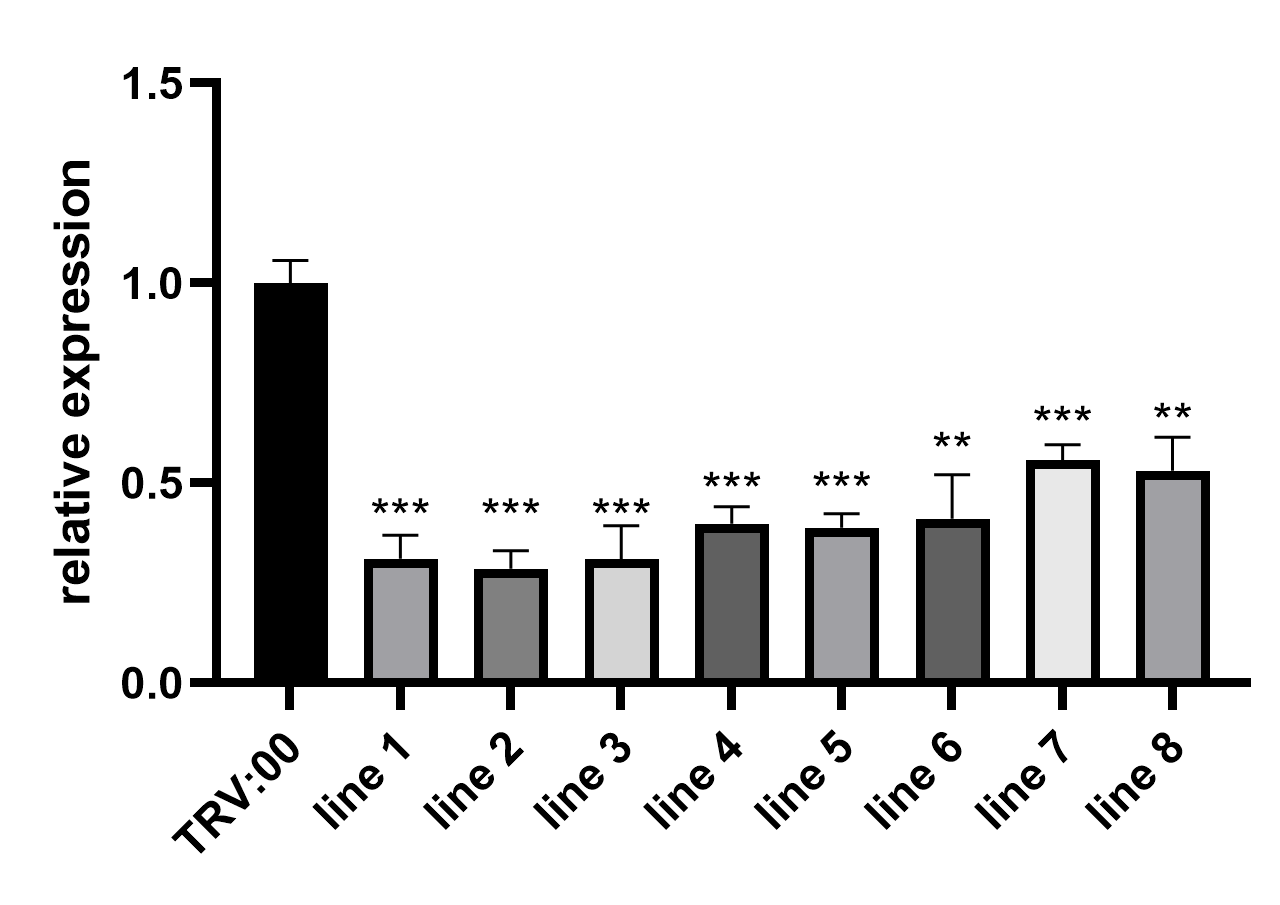

Supplement: Supplementary file 1 [file DataSheet1.ZIP › Fig S2.tif]
